# The management of common recurrent headaches by chiropractors: a descriptive analysis of a nationally representative survey

**DOI:** 10.1186/s12883-018-1173-6

**Published:** 2018-10-17

**Authors:** Craig Moore, Andrew Leaver, David Sibbritt, Jon Adams

**Affiliations:** 1 Faculty of Health, University of Technology Sydney, Level 8, Building 10, 235-253 Jones Street Ultimo, Sydney, NSW 2007 Australia; 2 Faculty of Health Science, University of Sydney, Sydney, Australia

**Keywords:** Chiropractic, Migraine, Tension headache, Cervicogenic headache, Manual therapy, Practice-based research network, Spinal manipulation

## Abstract

**Background:**

Headache management is common within chiropractic clinical settings; however, little is yet known about how this provider group manage headache sufferers. The aim of this study is to report on the prevalence of headache patients found within routine chiropractic practice and to assess how chiropractors approach key aspects of headache management applicable to primary care settings.

**Methods:**

A 31-item cross-sectional survey was distributed to a national sample of chiropractors (*n* = 1050) to report on practitioner approach to headache diagnosis, interdisciplinary collaboration, treatment and outcome assessment of headache patients who present with recurrent headache disorders.

**Results:**

The survey attracted a response rate of 36% (*n* = 381). One in five new patients present to chiropractors with a chief complaint of headache. The majority of chiropractors provide headache diagnosis for common primary (84.6%) and secondary (90.4%) headaches using formal headache classification criteria. Interdisciplinary referral for headache management was most often with CAM providers followed by GPs. Advice on headache triggers, stress management, spinal manipulation, soft tissue therapies and prescriptive neck exercises were the most common therapeutic approaches to headache management.

**Conclusion:**

Headache patients make up a substantial proportion of chiropractic caseload. The majority of chiropractors managing headache engage in headache diagnosis and interdisciplinary patient management. More research information is needed to understand the headache types and level of headache chronicity and disability common to chiropractic patient populations to further assess the healthcare needs of this patient population.

**Electronic supplementary material:**

The online version of this article (10.1186/s12883-018-1173-6) contains supplementary material, which is available to authorized users.

## Background

Tension headache and migraine are the most common recurrent primary headaches globally [1] and cervicogenic headache is one of the most common recurrent secondary headaches [2, 3]. While less information is available regarding the burden and economic impact associated with cervicogenic headache [4, 5], the societal impact of tension headache and migraine are significant and well documented [6–8].

In the collaborative study between the World Health Organisation (WHO) and the ‘*Lifting The Burden*’ campaign, survey information was collected from neurologists and general practitioners in order to better understand how these providers approach headache diagnosis and management [9]. The findings of the report provided important insights into the use of headache diagnostic criteria, headache assessment tools, headache treatment and interdisciplinary collaboration. While headache is most often managed by general practitioners and neurologists, the report also found headache patients report a clear preference for the use of complementary and alternative treatments for headaches including physical based therapies and acupuncture.

The use of chiropractors for headache management appears to be significant. In a recent national US study, manipulative-based physical therapies were reported to be the most frequently used complementary and alternative treatments for migraine and headache patients [10]. In North America, a general population study reported between 25.7–36.2% of migraine headache patients had sought help from chiropractors at some time [11]. In Australia, chiropractic utilisation by those with headache was reported to be 9.3% in the preceding 12 months [12]. Notably, one international study found chiropractors to be the second and third most common health care provider by those with migraine in Australia and the United States respectively [13].

While the use of chiropractors for the management of headache disorders appears to be significant, little is understood about how this provider group manage this substantial patient population. With increasing research examination on interdisciplinary headache management [14, 15], more information is needed to understand the role of chiropractors within the interdisciplinary headache management landscape. Gathering this information can offer important insights that may help to guide more effective and coordinated healthcare delivery between providers and improve the management of headache patients. In direct response to this important research gap, this paper reports on a) the prevalence of patients who present to chiropractors with headache and b) how chiropractors approach keys aspects of headache patient management appropriate to primary care settings including the use of headache diagnostic criteria, headache assessment tools, approach to headache treatment and interdisciplinary engagement with other headache providers.

## Methods

The study collected data via an online cross-sectional survey (Additional file 1) distributed to Australian practicing chiropractors who were recruited members of the Australian Chiropractic Research Network (ACORN) - a national practice-based research network (PBRN) [16]. Those recruited to the ACORN PBRN database are broadly representative of the wider national population of Australian chiropractors in terms of the key indicators of gender distribution, age distribution and practice location [17]. Full details of the original recruitment of chiropractors to join the national-based ACORN PBRN has been reported elsewhere [16]. This ACORN PBRN sub-study was approved by the Human Research Ethics Committee at the University of Technology Sydney (Approval number: ETH16–0639).

### Recruitment and participants

Practitioner recruitment for the sub-study was a random sample of chiropractors taken from the nationally representative ACORN database. A sample of 1050 participants was selected using the random number generator function in Microsoft Excel 2016. Recruitment was conducted between August and November 2016 with participants invited to complete a 31-item online headache questionnaire using the SurveyMonkey™ platform. An embedded link to the headache questionnaire was emailed to invited participants who received three reminders during the recruitment period.

### Instrument

The questionnaire introduction explained the approximate duration, purpose and contents of the study and that survey completion was voluntary, and that respondent information was anonymous. Consent was implied by completing the survey and no incentives were offered to participate in the study. As there are no previously validated instruments for the assessment of provider headache management across several clinical areas, the key themes and questions adopted for our study questionnaire were developed after consideration of the ‘WHO: Lifting the Burden’ report [9] and other surveys examining primary care management of headache patients [18, 19]. The headache disorders selected for the study were based upon headache types previously reported as common to chiropractic headache patient populations [20–22].

The questionnaire collected information on practitioner characteristics (i.e. gender, years in practice, place of education and practice location). Practitioner reporting of headache patient prevalence were based on practitioner consultations over the previous two weeks. Questions about the use of headache diagnostic criteria were based on the International Classification of Headache Disorders (ICHD-3 Beta) criteria for primary and secondary recurrent headaches [23]. Preceding the questions on primary headaches, the online questionnaire provided a direct link to ICHD-3 Beta diagnostic criteria. Preceding the questions on secondary headaches, a direct link was similarly provided to the ICHD-3 Beta diagnostic criteria. Questions regarding the use of headache assessment instruments were based on the use of the Migraine Disability Assessment questionnaire (MIDAS) [24], Headache Disability Inventory (HDI) [25] and the use of patient headache diaries [26]. For headache management, the questionnaire included questions on multi-disciplinary engagement with other providers (sending and receiving headache patient referrals) and questions on chiropractor’s approach to headache management including treatment aims, therapeutic methods and treatment volume. For questions regarding headache management by chiropractors, headaches were divided into headaches of less than 3 months’ duration and headaches of more than 3 months’ duration.

The questionnaire was pilot tested with 10 chiropractors in private clinical practice from different socio-demographic backgrounds who provided feedback on content, wording and survey length. Feedback from pilot testing resulted in further changes to the length and wording of the instrument. The final version of the online survey was estimated to take around 15 min to complete. All questionnaire items were either dichotomous (yes/no) or reported as ratings on a 4-point or 5-point Likert scale.

### Statistical analyses

Participant perceptions regarding the role of ICHD diagnostic criteria for primary and secondary headaches are re-categorized into 3 groups: strongly disagree/disagree; neutral and agree/strongly agree and the reporting of participant collaboration with other healthcare providers for the management of headache are re-categorised into 2 groups: never/rarely; and sometimes/often. This was due to the very low number of responses reported within some of the Likert categories provided for these questions. A minimum mean agreement score is used to report participant headache treatment aims (very unimportant/somewhat unimportant/neutral/somewhat important/very important). The reporting of chiropractic headache management provided by chiropractors are categorized as: often/almost every headache patient compared to never/rarely. Descriptive statistics are used to describe responses by participants. Continuous descriptive data are presented using means and standard deviations and categorical data presented using numbers and percentages. Statistical analysis was based upon the total number of completed surveys (*n* = 321) and conducted using software Stata 14.2.

## Results

### Practitioner characteristics

The questionnaire was completed by 381 practitioners, giving a response rate of 36.2%. This number represents 12.1% of the total number of practicing chiropractors in Australia at the time of recruitment. Participants mean number of years in practice was 18.1 years (SD = 10.9). When comparing survey participants to the ACORN data-base, survey respondents are generally representative for gender (64% male vs 63%) (*p* = 0.379), and place of practice: New South Wales (35.1% vs 34%), Victoria (23.2% vs 25%), Queensland (15.2% vs 15.0%), Western Australia (14.7% vs 13%), South Australia (8.5% vs 9.0%), Australian Capital Territory (1.6% vs 2%), Tasmania (0.9% vs 1%) and Northern Territory (0.5% vs 1%) (*p* = 0.916) [16]. These non-significant *p* values show no difference in distributions between samples for gender and place of practice, suggesting survey respondents are generally representative of the ACORN database participants. The distribution of these participant demographic characteristics are consistent with national registration records reported by the Chiropractic Board of Australia [27].

### Headache prevalence

In the previous two-week period the mean total number of new consultations reported by participants was 7.1 (SD = 4.8) where a chief complaint of headaches accounted for 1.5 (SD = 1.7) new consultations and a secondary complaint of headaches accounted for 2.5 (SD = 2.3) new consultations. In the previous two-week period the mean number of total patient consultations (new and routine treatment visits) was 170.9 (SD = 107.3) where a chief complaint of headaches accounted for 21.5 (SD = 28.6) total consultations and a secondary complaint of headaches accounted for 28.2 (33.8) total consultations.

### Headache treatment plans

In terms of the number of initial treatment visits normally provided for a new patient presenting with headaches of less than 3 months duration for each of migraine, tension headache and cervicogenic headache, between 28 and 29.6% of participants reported providing less than 5 treatments, 54.2–55.5% provided between 5 and 10 visits and 14.9–16.5% reported providing more than 10 visits across all 3 headache types. For the duration of an initial headache treatment plan for a new patient presenting with headaches of less than 3 months duration - migraine, tension headache and cervicogenic headache (grouped); 11.8% of participants reported providing treatment for less than 2 weeks, 50.3% reported 2–4 weeks, 33.0% reported 4–8 weeks and 4.4% reported treatment for more than 8 weeks. With regards to the frequency of treatment during an initial headache treatment plan for a new patient presenting with headaches of less than 3 months duration (i.e. migraine, tension headache and cervicogenic), 16.0% of participants reported providing one treatment per week, 72.5% two treatments per week, 11.0% three treatments per week and 0.5% reported providing more than three visits per week. In terms of the number of initial treatment visits for a new patient presenting with headaches for more than 3 months duration for each of migraine, tension headache and cervicogenic headache, between 10.7–12.0% of participants reported providing less than 5 treatments, 46.3–50.3% provided between 5 and 10 visits and between 38.0–43.0% reported providing more than 10 visits across all 3 headache types. For the duration of an initial headache treatment plan for a patient presenting with headaches for more than 3 months duration - migraine, tension headache and cervicogenic headache (grouped), 4.7% of participants reported providing treatment for less than 2 weeks, 32.2%% reported 2–4 weeks, 46.9% reported 4–8 weeks and 16.2% reported an initial treatment period of more than 8 weeks.

### Headache classification

**Fig. 1:**
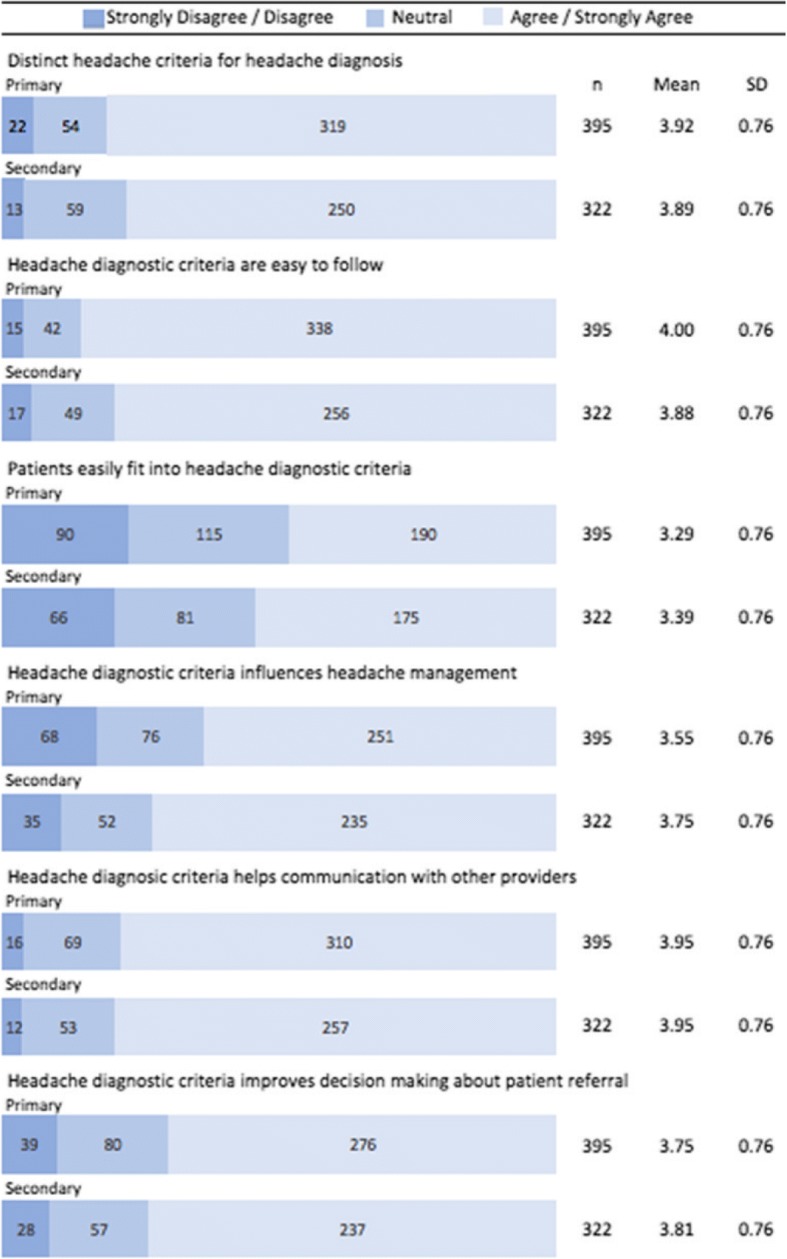
Chiropractors views regarding ICHD diagnostic criteria for primary and secondary headaches (strongly disagree/disagree/neutral/agree/strongly agree)

The majority of participants reported being familiar with ICHD headache criteria for primary (98.3%; *n* = 411) and secondary (81.2%; *n* = 324) headaches and using these criteria for classifying primary (84.6%; *n* = 334) and secondary (90.4%; *n* = 291) headaches. Figure 1 provides the mean score for participants’ perceptions regarding ICHD criteria for the diagnosis and management of primary and secondary headaches independently. The mean scores (0 = no agreement, 5 = high agreement) across all domains were high for participant agreement on the clinical utility of ICHD classification for a range of listed clinical purposes. There was a strong agreement amongst participants that ICHD criteria were easy to follow for primary (mean = 4.00; SD = 0.76) and secondary headaches (mean = 3.88; SD = 0.76) and represent distinct criteria for primary (mean = 3.92; SD = 0.76) and secondary headaches (mean = 3.89; SD = 0.76) and helps communication with other providers for primary (mean = 3.95; SD = 0.76) and secondary headaches (mean = 3.96; SD = 0.76). There was relatively less agreement amongst participants that patients easily fit into ICHD criteria for primary (mean = 3.29; SD = 0.76) and secondary headaches (mean = 3.39; SD = 0.76).

### Multidisciplinary care

The level of interdisciplinary collaboration between chiropractors and other healthcare providers in managing patients with headaches is reported in Table 1. The most frequent collaboration between chiropractors and other providers for headache management was reported to be with other Complementary and Alternative Medicine (CAM) providers, followed by GPs for both referring and receiving headache patient referrals. The frequency of chiropractors referring headache patients to GPs was reported as substantially higher than the frequency of chiropractors receiving headache patient referrals from GPs.

**Table 1:** Interdisciplinary collaboration by chiropractors with other healthcare providers for headache management (sometimes/often compared to never/rarely)

Provider	Receiving (sometimes/often) *n* = 392	Referring (sometimes/often) *n* = 392
CAM practitioner	66.1% (*n* = 259)	66.3% (*n* = 260)
General practitioner	29.6% (*n* = 116)	59.9% (*n* = 235)
Medical specialist (via GP)	3.8% (*n* = 15)	42.6% (*n* = 167)
Dentist	25% (*n* = 98)	40.3% (*n* = 158)
Psychologist	10.9% (*n* = 43)	16.6% (*n* = 65)
Physiotherapist	11.7% (*n* = 46)	13.3% (*n* = 52)
Osteopath	5.3% (n = 21)	3.8% (n = 15)

Survey key: Medical specialist (via GP) e.g. neurologist, psychiatrist. CAM practitioner e.g. acupuncturist, herbalist, naturopath, massage therapist, counsellor

The reasons chiropractors ‘sometimes’ or ‘often’ refer headache patients to other providers was to: investigate headache red-flags (83.4%; *n* = 324); assist with acute headache pain (57.1%; *n* = 224); assist with headache-related coping skills (53.8%; *n* = 211); assist with headache prevention (44.9%; *n* = 176); and confirm headache diagnosis (32.9%; *n* = 129).

### Chiropractic headache management

The mean scores (0 = no agreement, 5 = high agreement) across all domains were high for participant agreement on the importance of a range of headache treatment outcomes. There was a minimum mean agreement score of 4.23 out of 5 for: the importance of treatment providing headache prevention; improving headache recovery and headache pain relief; improving headache-related coping skills; and patient health and well-being.

The most frequent therapeutic approach by participants for migraine management was advice on headache triggers (94.1%), stress management (89.4%) and non-thrust spinal mobilisation (88.4%). The most frequent therapeutic approach by participants for tension headache management was advice on headache triggers (90.9%), stress management (90.1%) and soft tissue therapies (massage, myofascial, stretching or trigger point therapy) to the neck/shoulder area (88.1%). The most frequent therapeutic approach by participants for cervicogenic headache management was prescription exercises for the neck/shoulders (91.7%), spinal manipulation (90.6%) and soft tissue therapies (massage, myofascial, stretching or trigger point therapy) to the neck/shoulder area (88.3%) (Table 2).

**Table 2:** Headache management characteristics by chiropractors (often/almost every headache patient compared to never/rarely)

Treatment approach	Migraine (often/almost all) (*n* = 387)	Tension headache (often/almost all) (*n* = 382)	Cervicogenic headache (often/almost all) (n = 382)
Joint-based manipulative therapies			
Spinal manipulation	318(82.2%)	337(87.5%)	349(90.6%)
Non-thrust spinal mobilisations	264(88.4%)	252(65.5%)	252(65.5%)
Instrument adjusting	279(72.1%)	270(70.1%)	273(70.9%)
Drop-piece methods	133(34.4%)	148(38.4%)	153(39.7%)
Soft-tissue based and exercise therapies			
Soft tissue to neck/shoulders	331(85.3%)	339(88.1%)	340(88.3%)
Electro-physical therapies	30(7.8%)	30(7.8%)	30(7.8%)
Soft-tissue/exercise to temporomandibular	252(65.1%)	249(64.7%)	233(60.5%)
Exercises – neck/shoulders	311(81.6%)	337(87.5%)	353(91.7%)
Patient advice and education			
Advice on headache triggers	364(94.1%)	350(90.9%)	338(87.8%)
Advice on diet and fitness	331(85.6%)	336(87.3%)	327(84.9%)
Stress management	346(89.4%)	347(90.1%)	337(87.5%)

Survey key: Spinal manipulation (manual adjusting/manipulation (including Diversified, Gonstead); Drop piece methods (drop-piece/Thompson or similar); Soft tissue – neck/shoulders (massage, myofascial, stretching or trigger points to neck/shoulders); Electro-physical therapies (including TENS, ultrasound)

When asked about the use of headache assessment instruments, a significant percentage of participants reported ‘never’ or ‘rarely’ using MIDAS (96.2%) and HDI (87.3%) headache instruments. The use of headache diaries was reported as ‘sometimes’ or ‘almost every headache patient’ by 41% of the chiropractors (data not shown).

## Discussion

Results from our study suggest that a large percentage of new and routine chiropractic patient consultations are related to headache management with around one in five new patients presenting to chiropractors with a chief complaint of headache and more than one in three presenting with a secondary complaint of headache. This substantial level of headache caseload within chiropractic clinical settings raises questions about the factors that influence the preference and use of chiropractors for the management of headache compared to the use of other headache providers and treatments. Previous evidence suggests that patient dissatisfaction with preventative headache drug treatments are likely to be an important predictor for headache patient use of manual therapy providers [21]. However, there is a need for more robust research to assess the effectiveness of manual therapies for the prevention of recurrent headaches. To date, systematic reviews report significant methodological short-comings for clinical trials that aim to assess the prevention of migraine with manual therapies [28, 29], while limited, moderate quality evidence appears to support the potential role of manual therapies for the prevention of tension-type headache [30, 31] and cervicogenic headache [32, 33].

Our study results suggest some aspects of headache patient management by chiropractors are consistent with that of medical providers. For example, the proportion of chiropractors reporting the use of primary and secondary headache diagnostic criteria in our study (84.6% and 90.4% respectively) compares favourably with the use of headache diagnosis found within medical care [9, 18]. While headache diagnosis is likely to improve clinical decision-making when managing the healthcare needs of headache sufferers [34], there is currently limited, poor quality information reporting on the proportion of migraine [13], tension headache [22], and cervicogenic headache within chiropractic clinical settings. As such, more information is needed to better understand the types of headaches and level of headache burden more common to chiropractic clinical settings and how the management of headache patients is influenced by headache diagnosis including approaches to patient examination, education, referral and treatment.

Of note, practitioner use of secondary headache criteria for cervicogenic and medication over-use headache was reported slightly more often than practitioner familiarity with these secondary headache criteria. Poor familiarity with secondary headache criteria raises concerns about the risk to patient outcomes should chiropractors fail to appropriately diagnose secondary headaches. Such concerns could have serious consequences for secondary headaches needing urgent medical management [35]. While fully understanding this finding requires further empirical investigation, another explanation may be that some chiropractors are less familiar with at least some secondary headache diagnostic criteria listed, a finding that may relate to medication overuse headache, a secondary headache condition that can go unrecognized in clinical settings [36]. Additionally, this finding may also relate to practitioner concerns regarding the clinical utility of the diagnostic criteria associated with cervicogenic headache, an issue that has been reported elsewhere [3, 37, 38]. If so, these results may add weight to the need for further research examination into provider understanding, use and acceptance of cervicogenic headache criteria within primary care clinical settings.

The high rate of headache referral (receiving/referring) between chiropractors and other CAM providers in our study is consistent with findings from previous research in Australia and the US [39, 40]. The pattern of high referral between chiropractors and other CAM providers may be influenced by a number of factors including the influence of chiropractic organisations who sometimes promote a drugless approach to patient care [41, 42] or the higher percentage of chiropractors working at the same practice location as other CAM providers when compared to those practicing alongside other healthcare providers [40].

Our study identified that less than one in three chiropractors sometimes or often receive headache referrals from GPs. While the implication of these findings requires further empirical inquiry, this low rate of headache referral from GPs may be due to factors including GP concerns about the current level of evidence to support the effectiveness of manual therapies for the management of headache or a less favourable GP attitude toward chiropractors as reported in a recent survey which found that 60% of Australian GPs never referred patients to a chiropractor [43]. With systematic reviews reporting evidence to support the potential role of manual therapies for some headache types [31, 32, 44], further research may be needed to better understand the current barriers to collaborative headache management that may exist between these providers. This research priority would seem important given the unmet needs remaining for some headache sufferers under medical care [45–48] and the high use of manipulative therapy providers by headache patients [10, 13, 21].

While the low frequency of headache patient referral between chiropractors and physiotherapy and osteopathic providers in our study may be partly explained by the use of similar approaches to headache treatment [49, 50], the low frequency of headache patient referral between chiropractors and psychologists deserves further consideration. Psychologists are a significant healthcare provider for the management of headache pain [51, 52] and for the management of headache-related comorbidities such as anxiety and depression. [53, 54]. As such, this finding raises questions about whether chiropractors managing headache are fully aware of the psycho-behavioural approaches available to assist in the management of headache. In comparison, the higher frequency of headache patient referral to GPs and medical specialists (via the GP) by chiropractors appears to suggest there are circumstances where chiropractors are working together with medical providers for the management of headache, a finding further supported by the high frequency of referral for the investigation of headache red-flags reported in our study. More information reporting on the types of headaches, level of headache chronicity and disability found within chiropractic headache populations would further help researchers and clinicians to better comprehend the related healthcare needs of this patient population and the clinical circumstances where greater interdisciplinary collaboration is warranted between chiropractors and other headache-related healthcare providers.

The most common therapeutic approaches reported by chiropractors in our study for the management of headache was providing advice on headache triggers, stress management, spinal manipulation, soft tissue therapies and prescriptive neck exercises. Helping patients both identify and manage headache triggers is recognised as an important aspect of headache patient management for those who present with migraine and tension headache within primary care settings [55]. However, the role of manual and exercise therapies for the management of those with recurrent headaches remains less certain with systematic reviews reporting stronger evidence for manual therapies for the prevention of cervicogenic and tension headache [31, 32] and limited and conflicting evidence for the prevention of migraine [29]. As such, more robust research is needed to assess the effectiveness of both unimodal and multi-modal approaches to headache management by chiropractors, including for the management of both acute and chronic headache sub-types.

The chiropractors in our study most often provided between 5 and 10 treatments during an initial headache treatment plan while a slightly higher average number of treatments were provided for those with headaches of longer duration (more than 3 months). This number of treatments is similar to the number of treatments associated with significant improvement in headache outcomes for spinal mobilisation and manipulation reported in previous tension headache and cervicogenic headache studies [56, 57]. While information is limited regarding the relative costs associated with chiropractic headache management, one recent US study compared the cost of headache care using risk-adjusted scores that would otherwise affect the level of healthcare utilization [58]. This study found headache treatment costs were significantly higher both for medical doctor-only care when compared to chiropractic-only care and for medical doctor care combined with physical therapy care compared to medical doctor care combined with chiropractic care.

Our study found chiropractors more frequently engage the use of patient headache diaries, an approach to headache assessment that can help to reduce patient difficulty in recalling headache characteristics and their response to headache treatment [59]. However, the use of formal headache instruments such as MIDAS and HDI was comparatively low, a finding reported within other primary care settings [9, 60]. These validated headache instruments can assist health care providers to better understand headache disability, exacerbations and remissions and circumstances that indicate the need for specialty care [25, 61, 62]. As such, the low use of validated headache instruments reported in our study raises questions about best practice with regards to chiropractors more fully assessing headache patients to better understand clinical findings associated with more complex headache patient presentations.

A key strength of our study is the nationally representative cross-sectional sample of chiropractors in order to provide important preliminary information on the current state of chiropractic headache practice. It is however important to acknowledge several limitations to our study. While the online survey provided a direct reference and link to the ICHD-3 classification criteria for primary and secondary headaches, a comprehensive list of the headache criteria was not provided within the survey prior to asking respondents if they were familiar with the diagnostic criteria for the primary and secondary headaches listed. Furthermore, the survey has not aimed to explore diagnosis and management of chronic headache types (15 or more days per month over a 3-month period). The response rate for our sample (36%), while similar to other studies of this type, is limited to 12% of the total practitioner population nationally. As a result, there may be important differences in the headache management characteristics between survey respondents and non-respondents. This would include the risk of selection bias that may result from the random selection of chiropractors within a PBRN compared to outside the PBRN. The Likert categories utilized in parts of the survey questionnaire are open to practitioner interpretation and findings are based upon self-report and retrospective recall and subject to recall bias. In addition, our study did not provide any assessment of adverse events that may result from manual therapies for the management of headaches. However, these findings draw upon a national sample of chiropractors in order to provide valuable insights for future investigation to further our understanding of the management of headache patients by this provider group.

## Conclusions

Our national-based sample suggests headache is a substantial proportion of chiropractic caseload. While some aspects of chiropractic headache management, including the acceptance and use of headache diagnostic criteria, appears to be consistent with good clinical practice, other aspects of chiropractic headache management raise questions worthy of further research enquiry. Critically, there is a need for more detailed information on the proportion of headache types and level of headache chronicity and disability found within chiropractic headache patient populations. This information will help practitioners, researchers and policy-makers to better understand the healthcare needs associated with headache patients who seek help from this common provider of headache management.

## Additional file

Additional file 1: Survey questionnaire. (PDF 2893 kb)

## Abbreviations

ACORN: Australian chiropractic research network
CAM: Complementary and alternative medicine
HDI: Headache disability inventory
ICHD: International classification of headache disorders
MIDAS: Migraine disability assessment questionnaire
PBRN: Practice-based research network

## References

[1] Vos T, Flaxman AD, Naghavi M, Lozano R, Michaud C, Ezzati M (2012). Years lived with disability (YLDs) for 1160 sequelae of 289 diseases and injuries 1990–2010: a systematic analysis for the global burden of disease study. Lancet.

[2] Sjaastad O, Fredriksen T (2000). Cervicogenic headache: criteria, classification and epidemiology. Clin Exp Rheumatol.

[3] Sjaastad O, Bakketeig LS (2008). Prevalence of cervicogenic headache: Vågå study of headache epidemiology. Acta Neurol Scand.

[4] HAv S, Lamé I, van den Berg SGM S, AGH K, WEJ W (2003). Quality of life of patients with cervicogenic headache: a comparison with control subjects and patients with migraine or tension-type headache. Headache.

[5] Gesztelyi G, Bereczki D (2006). Determinants of disability in everyday activities differ in primary and cervicogenic headaches and in low back pain. Psychiatry Clin Neurosci.

[6] Zebenholzer K, Andree C, Lechner A, Broessner G, Lampl C, Luthringshausen G (2015). Prevalence, management and burden of episodic and chronic headaches—a cross-sectional multicentre study in eight Austrian headache centres. J Headache Pain.

[7] Lanteri-Minet M (2014). Economic burden and costs of chronic migraine. Curr Pain Headache Rep.

[8] Yu S, Han X (2014). Update of chronic tension-type headache. Curr Pain Headache Rep.

[9] WHO Lifting the Burden [http://www.who.int/mental_health/management/who_atlas_headache_disorders.pdf?ua=1]. Accessed 1 Sept 2018.

[10] Zhang Y, Dennis JA, Leach MJ, Bishop FL, Cramer H, Chung VC (2017). Complementary and alternative medicine use among US adults with headache or migraine: results from the 2012 National Health Interview Survey. Headache.

[11] Bigal ME, Serrano D, Reed M, Lipton RB (2008). Chronic migraine in the population burden, diagnosis, and satisfaction with treatment. Neurology.

[12] Xue C, Zhang A, Lin V, Myers R, Polus B, Story D (2008). Acupuncture, chiropractic and osteopathy use in Australia: a national population survey. BMC Public Health.

[13] Sanderson JC, Devine EB, Lipton RB, Bloudek LM, Varon SF, Blumenfeld AM (2013). Headache-related health resource utilisation in chronic and episodic migraine across six countries. J Neurol Neurosurg Psychiatry.

[14] Nicol AL, Hammond N, Doran SV (2013). Interdisciplinary management of headache disorders. Tech Reg Anesth Pain Manag.

[15] Gaul Charly, Liesering-Latta Eva, Schäfer Benjamin, Fritsche Günther, Holle Dagny (2016). Integrated multidisciplinary care of headache disorders: A narrative review. Cephalalgia.

[16] Adams J, Steel A, Moore C, Amorin-Woods L, Sibbritt D (2016). Establishing the ACORN National Practitioner Database: strategies to recruit practitioners to a National Practice-Based Research Network. J Manip Physiol Ther.

[17] Adams J, Peng W, Steel A, Lauche R, Moore C, Amorin-Woods L (2017). A cross-sectional examination of the profile of chiropractors recruited to the Australian chiropractic research network (ACORN): a sustainable resource for future chiropractic research. BMJ Open.

[18] Kernick D, Stapley S, Hamilton W (2008). GPs’ classification of headache: is primary headache underdiagnosed?. Br J Gen Pract.

[19] Vuillaume De Diego E, Lanteri-Minet M (2005). Recognition and management of migraine in primary care: influence of functional impact measured by the headache impact test (HIT). Cephalalgia.

[20] Adams J, Barbery G, Lui C-W (2013). Complementary and alternative medicine use for headache and migraine: a critical review of the literature. Headache.

[21] Moore CS, Sibbritt DW, Adams J (2017). A critical review of manual therapy use for headache disorders: prevalence, profiles, motivations, communication and self-reported effectiveness. BMC Neurol.

[22] Kristoffersen ES, Grande RB, Aaseth K, Lundqvist C, Russell MB (2012). Management of primary chronic headache in the general population: the Akershus study of chronic headache. J Headache Pain.

[23] Headache Classification Committee of the International Headache S (2013). The international classification of headache disorders, 3rd edition (beta version). Cephalalgia.

[24] Stewart Walter F., Lipton Richard B., Kolodner Kenneth B., Sawyer James, Lee Clara, Liberman Joshua N. (2000). Validity of the Migraine Disability Assessment (MIDAS) score in comparison to a diary-based measure in a population sample of migraine sufferers. Pain.

[25] Jacobson GP, Ramadan NM, Aggarwal SK, Newman CW (1994). The Henry ford hospital headache disability inventory (HDI). Neurology.

[26] Phillip D, Lyngberg A, Jensen R (2007). Assessment of headache diagnosis. A comparative population study of a clinical interview with a diagnostic headache diary. Cephalalgia.

[27] Chiropractic Board of Australia. Chiropractic registrant data [http://www.chiropracticboard.gov.au/About-the-Board/Statistics.aspx]. Accessed 1 Oct 2018.

[28] Chaibi A, Tuchin PJ, Russell MB (2011). Manual therapies for migraine: a systematic review. J Headache Pain.

[29] Posadzki P, Ernst E (2011). Spinal manipulations for the treatment of migraine: a systematic review of randomized clinical trials. Cephalalgia.

[30] Lozano López C, Mesa Jiménez J, de la Hoz Aizpurúa JL, Pareja Grande J, Fernández de las Peñas C (2016). Efficacy of manual therapy in the treatment of tension-type headache. A systematic review from 2000 to 2013. Neurología (English Edition).

[31] Mesa-Jiménez JA, Lozano-López C, Angulo-Díaz-Parreño S, Rodríguez-Fernández ÁL, De-la-Hoz-Aizpurua JL, Fernández-de-las-Peñas C (2015). Multimodal manual therapy vs pharmacological care for management of tension type headache: a meta-analysis of randomized trials. Cephalalgia.

[32] Racicki S, Gerwin S, DiClaudio S, Reinmann S, Donaldson M (2013). Conservative physical therapy management for the treatment of cervicogenic headache: a systematic review. J Man Manip Ther.

[33] Chaibi A, Russell MB (2012). Manual therapies for cervicogenic headache: a systematic review. J Headache Pain.

[34] Kingston WS, Halker R. Determinants of suboptimal migraine diagnosis and treatment in the primary care setting. JCOM. 2017;24:319–24.

[35] Sarah N, TL P (2014). Headaches in brain tumor patients: primary or secondary?. Headache.

[36] Obermann M, Katsarava Z (2007). Management of medication-overuse headache. Expert Rev Neurother.

[37] Fredriksen T, Antonaci F, Sjaastad O (2015). Cervicogenic headache: too important to be left un-diagnosed. J Headache Pain.

[38] Antonaci F, Bono G, Chimento P (2006). Diagnosing cervicogenic headache. J Headache Pain.

[39] Pohlman KA, Hondras MA, Long CR, Haan AG (2010). Practice patterns of doctors of chiropractic with a pediatric diplomate: a cross-sectional survey. BMC Complement Altern Med.

[40] Adams J, Lauche R, Peng W, Steel A, Moore C, Amorin-Woods LG (2017). A workforce survey of Australian chiropractic: the profile and practice features of a nationally representative sample of 2,005 chiropractors. BMC Complement Altern Med.

[41] Chiropractic and you. https://chiropractors.asn.au/about-chiropractic/chiropractic-and-you. Accessed 1 Oct 2018.

[42] World Federation of Chiropractic Identity Task Report [https://www.wfc.org/website/images/wfc/docs/as_tf_final_rept-Am_04-29-05_001.pdf]. Accessed 3 Jan 2017.

[43] Engel RM, Beirman R, Grace S (2016). An indication of current views of Australian general practitioners towards chiropractic and osteopathy: a cross-sectional study. Chiropr Man Therap.

[44] Chaibi A, Russell MB (2014). Manual therapies for primary chronic headaches: a systematic review of randomized controlled trials. J Headache Pain.

[45] Rossi P, Di Lorenzo G, Faroni J, Malpezzi MG, Cesarino F, Nappi G (2006). Use of complementary and alternative medicine by patients with chronic tension-type headache: results of a headache clinic survey. Headache.

[46] Gaul C, Eismann R, Schmidt T, May A, Leinisch E, Wieser T (2009). Use of complementary and alternative medicine in patients suffering from primary headache disorders. Cephalalgia.

[47] Malone CD, Bhowmick A, Wachholtz AB (2015). Migraine: treatments, comorbidities, and quality of life, in the USA. J Pain Res.

[48] Starling Amaal J., Dodick David W. (2015). Best Practices for Patients With Chronic Migraine. Mayo Clinic Proceedings.

[49] Grant T, Niere K (2000). Techniques used by manipulative physiotherapists in the management of headaches. Aust J Physiother.

[50] Schabert E, Crow WT (2009). Impact of osteopathic manipulative treatment on cost of care for patients with migraine headache: a retrospective review of patient records. J Am Osteopath Assoc.

[51] Bendtsen L, Evers S, Linde M, Mitsikostas DD, Sandrini G, Schoenen J (2010). EFNS guideline on the treatment of tension-type headache – report of an EFNS task force. Eur J Neurol.

[52] Pringsheim T, Davenport WJ, Mackie G, Worthington I, Aubé M, Christie SN (2012). Canadian headache society guideline for migraine prophylaxis. Can J Neurol Sci.

[53] Seng EK, Mayson SJ, Sonty N, Stein T, Tsui P, Qian S (2014). Psychological considerations in the migraine patient. Migraine Surgery edn.

[54] Jensen R, Zeeberg P, Dehlendorff C, Olesen J (2010). Predictors of outcome of the treatment programme in a multidisciplinary headache centre. Cephalalgia.

[55] Hoque B, Rahman KM, Hasan ATMH, Chowdhury RN, Khan SU, Alam MB, et al. Precipitating and relieving factors of migraine versus tension type headache. BMC Neurol. 2012;12:1–4. 10.1186/1471-2377-12-82 PMC3503560 22920541

[56] Haas Mitchell, Spegman Adele, Peterson David, Aickin Mikel, Vavrek Darcy (2010). Dose response and efficacy of spinal manipulation for chronic cervicogenic headache: a pilot randomized controlled trial. The Spine Journal.

[57] Castien RF, Windt DA, Grooten A, Dekker J (2011). Effectiveness of manual therapy for chronic tension-type headache: a pragmatic, randomised, clinical trial. Cephalalgia.

[58] Hurwitz EL, Vassilaki M, Li D, Schneider MJ, Stevans JM, Phillips RB (2016). Variations in patterns of utilization and charges for the Care of Headache in North Carolina, 2000-2009: a statewide claims data analysis. J Manip Physiol Ther.

[59] Jensen R, Tassorelli C, Rossi P, Allena M, Osipova V, Steiner T (2011). A basic diagnostic headache diary (BDHD) is well accepted and useful in the diagnosis of headache. A multicentre European and Latin American study. Cephalalgia.

[60] Minen MT, Loder E, Tishler L, Silbersweig D (2016). Migraine diagnosis and treatment: a knowledge and needs assessment among primary care providers. Cephalalgia.

[61] Stewart WF, Lipton RB, Kolodner K (2003). Migraine disability assessment (MIDAS) score: relation to headache frequency, pain intensity, and headache symptoms. Headache.

[62] Lipton RB, Stewart WF, Sawyer J, Edmeads JG (2001). Clinical utility of an instrument assessing migraine disability: the migraine disability assessment (MIDAS) questionnaire. Headache.

