# Supplementary material for: The management of common recurrent headaches by chiropractors: a descriptive analysis of a nationally representative survey
Source: BMC Neurol. 2018 Oct 17;18:171. doi: 10.1186/s12883-018-1173-6 (PMC6192187; doi:10.1186/s12883-018-1173-6)
Supplement: Supplementary file 1 — Survey questionnaire. (PDF 2893 kb) [file 12883_2018_1173_MOESM1_ESM.pdf]

ACORN
Australian Chiropractic Research Network

The management of headache disorders by Australian chiropractors

INFORMATION SHEET AND CONSENT FORM
Approval number: UTS HREC ETH16-0639

I am delighted to invite you to participate in this ACORN research sub-study. My name is Dr Craig Moore (Chiropractor) and I am a PhD candidate at the University of Technology Sydney. My principal supervisor is Professor Jon Adams (co-supervisors Professor David Sibbritt and Dr Andrew Leaver). This research is an opportunity for you to contribute to the evidence-base for chiropractic.

The purpose of this research is to find out more information about the management provided by Australian chiropractors for those with headache disorders. This online questionnaire will first ask a few questions about you, including your place of education, years in practice, followed by questions about your headache patient caseload and finishes with asking how you manage those with headaches, including your approach to diagnosis, collaboration and treatment.

Completing this questionnaire is voluntary. The information you provide will be anonymous and will only be accessed by the research team and stored in a fully secured software management system. This should take less than 15 minutes to complete. Your completion of the questionnaire implies consent. You may change your mind at any time and stop completing the questionnaire without consequence. If you stop and later return back to the survey your completed answers will be saved.

With the understanding that the information gathered can only be published in a form that does not identify you, please continue with answering the survey questions.

If you would like to talk to someone who is not connected with the research, you may contact the Research Ethics Officer on 02 9514 2478 or Research.ethics@uts.edu.au and quote this ethics approval number UTS HREC ETH16-0639

If you have concerns about the research that you think I or my supervisor can help you with, please feel free to contact the projects research assistant on 02-95148050 or email Jon.Adams@uts.edu.au

ACORN
Australian Chiropractic Research Network

The management of headache disorders by Australian chiropractors

Practitioner Demographics

This section is about you.

* 1. What is your gender?

- ◯ Male
- ◯ Female
- ◯ Other

* 2. How many years have you been in practice?

* 3. Please identify where you received your chiropractic education.

* 4. Where do you currently practice? (You may select more than one response)

- ☐ NSW
- ☐ VIC
- ☐ WA
- ☐ QLD
- ☐ TAS
- ☐ SA
- ☐ NT

The management of headache disorders by Australian chiropractors

Headache Prevalence

This section is about the number of patients you consulted in the LAST TWO WEEKS.

* 5. In the last two weeks, how many NEW patients did you consult?

* 6. In the last two weeks, how many NEW patients did you consult who had a chief complaint of headache?

* 7. In the last two weeks, how many NEW patients did you consult who had a secondary complaint of headache (i.e. headache present but not their chief complaint)?

* 8. In the last two weeks, how many consultations did you undertake in TOTAL?

* 9. In the last two weeks, how many consultations did you undertake where the chief complaint was headache?

* 10. In the last two weeks, how many consultations did you undertake where the secondary complaint was headache (i.e headache present but not the chief complaint)?

ACORN
Australian Chiropractic Research Network

The management of headache disorders by Australian chiropractors

Headache Classification

This section is about the diagnostic criteria you use for headaches.

Questions 11-13 relate to the diagnostic criteria provided below for **primary headache types** as recommended by the International Classification of Headache Disorders (ICHD) as per ihs-classification.org

Tension-type headache

Episodes of headache, typically bilateral, pressing or tightening in quality and of mild to moderate intensity, lasting minutes to days. The pain does not worsen with routine physical activity and is not associated with nausea, but photophobia or phonophobia may be present.

Migraine type headache

Recurrent headache disorder manifesting in attacks lasting 4-72 hours. Typical characteristics of the headache are unilateral location, pulsating quality, moderate or severe intensity, aggravation by routine physical activity and association with nausea and/or photophobia and phonophobia.

Cluster headache

Attacks of severe, strictly unilateral pain which is orbital, supraorbital, temporal or in any combination of these sites, lasting 15–180 minutes and occurring from once every other day to eight times a day. The pain is associated with ipsilateral conjunctival injection, lacrimation, nasal congestion, rhinorrhoea, forehead and facial sweating, miosis, ptosis and/or eyelid oedema, and/or with restlessness or agitation.

* 11. Are you familiar with these diagnostic criteria for these primary headaches?

◯ Yes

◯ No

ACORN
Australian Chiropractic Research Network

The management of headache disorders by Australian chiropractors

Headache Classification

Primary Headaches

* 12. Do you use these diagnostic criteria for primary headache types such as migraine, tension-type headache or cluster headache?

- ◯ Yes
- ◯ No

* 13. To what extent do you agree with the following statements about these criteria for the diagnosis of PRIMARY headaches.

	Strongly disagree	Disagree	Neutral	Agree	Strongly agree
These are distinct headache criteria for the diagnoses of primary headache types.	○	○	○	○	○
These diagnostic criteria for primary headache types are easy for me to follow.	○	○	○	○	○
My patients with primary headache easily fit into these diagnostic criteria.	○	○	○	○	○
These diagnostic criteria influence my management of patients with primary headaches.	○	○	○	○	○
Using these diagnostic criteria for primary headaches helps my communication with other healthcare professionals.	○	○	○	○	○
Using these diagnostic criteria improves my decision-making about patient referral or co-management for those with primary headaches.	○	○	○	○	○

The management of headache disorders by Australian chiropractors

Headache Classification

Secondary Headaches

Questions 14-16 relate to the diagnostic criteria provided below for **secondary headache types** as recommended by the International Classification of Headache Disorders (ICHD) as per ihs-classification.org

Cervicogenic headache

Headache caused by a disorder of the cervical spine and its component bony, disc and/or soft tissue elements, usually but not invariably accompanied by neck pain.

Medication overuse headache

Headache occurring on 15 or more days per month developing as a consequence of regular overuse of acute or symptomatic headache medication (on 10 or more, or 15 or more days per month, depending on the medication) for more than 3 months. It usually, but not invariably, resolves after the overuse is stopped.

* 14. Are you familiar with these diagnostic criteria for these secondary headaches?

- Yes
- No

The management of headache disorders by Australian chiropractors

Headache Classification

Secondary Headaches

* 15. Do you use these diagnostic criteria for secondary headache types such as cervicogenic or medication-overuse headache?

- Yes
- No

* 16. To what extent do you agree with the following statements about these headache diagnostic criteria for the diagnosis of SECONDARY headaches.

	Strongly disagree	Disagree	Neutral	Agree	Strongly agree
These are distinct headache criteria for the diagnoses of secondary headache types.	○	○	○	○	○
These diagnostic criteria for secondary headache types are easy for me to follow.	○	○	○	○	○
My patients with headache easily fit into these secondary headache categories.	○	○	○	○	○
These diagnostic criteria influence my management of patients with secondary headaches.	○	○	○	○	○
Using these diagnostic criteria for secondary headaches helps my communication with other healthcare professionals.	○	○	○	○	○
Using these diagnostic criteria improves my decision-making about patient referral or co-management for those with secondary headaches	○	○	○	○	○

ACORN
Australian Chiropractic Research Network

The management of headache disorders by Australian chiropractors

Headache Classification

Other labels

* 17. Do you use other labels or words to diagnose headache types other than those recommended by the ICHD classification (primary or secondary)?

- ◯ Yes
- ◯ No

ACORN
Australian Chiropractic Research Network

The management of headache disorders by Australian chiropractors

Headache Classification

Other labels

18. If so please list these labels/descriptions below:

1.

2.

3.

4.

5.

The management of headache disorders by Australian chiropractors

Treatment Outcome Measures

This section is about outcome measures you may use for monitoring headaches.

* 19. How often do you use the following outcome measures to monitor new patients who present with a chief complaint of headache?

	Never	Rarely	Often	Every new patient with headache
Migraine Disability Assessment Test (MIDAS)	○	○	○	○
Headache Disability Inventory (HDI)	○	○	○	○
Headache Diary	○	○	○	○

ACORN
Australian Chiropractic Research Network

The management of headache disorders by Australian chiropractors

Multidisciplinary care

These questions are about your collaboration with other health providers.

* 20. How often do you RECEIVE a patient referral from the following healthcare professionals for the management of headache?

	Never	Rarely	Sometimes	Often
General medical practitioner	◯	◯	◯	◯
Medical Specialist (eg neurologist, rheumatologist, orthopaedic, psychiatrist)	◯	◯	◯	◯
Psychologist	◯	◯	◯	◯
Dentist	◯	◯	◯	◯
Physiotherapist	◯	◯	◯	◯
Osteopath	◯	◯	◯	◯
CAM practitioners (including acupuncturist, herbalist, naturopath, massage therapist, counsellor)	◯	◯	◯	◯

* 21. How often do you REFER a patient to the following healthcare professionals for the management of headache?

	Never	Rarely	Sometimes	Often
General medical practitioner	◯	◯	◯	◯
Medical Specialist (via the GP) e.g neurologist, rheumatologist, orthopaedic, psychiatrist	◯	◯	◯	◯
Psychologist	◯	◯	◯	◯
Dentist	◯	◯	◯	◯
Physiotherapist	◯	◯	◯	◯
Osteopath	◯	◯	◯	◯
CAM practitioners (including acupuncturist, herbalist, naturopath, massage therapist, counsellor)	◯	◯	◯	◯

* 22. How often would you refer a patient with headache to another healthcare professional based on the following reasons?

	Never	Rarely	Sometimes	Often
To seek or confirm the headache classification/diagnosis	◯	◯	◯	◯
To improve the patient's coping skills and management of headache-related disability	◯	◯	◯	◯
To investigate a headache red-flag	◯	◯	◯	◯
To provide pain relief for acute headache attacks	◯	◯	◯	◯
To help provide headache prevention	◯	◯	◯	◯

ACORN
Australian Chiropractic Research Network

The management of headache disorders by Australian chiropractors

Multidisciplinary care

* 23. To what extent are you aware of the following indications for urgent medical attention in patients with headache (red-flags)?

	Not at all aware	Somewhat aware	Highly aware
Sudden onset of headache following head or neck injury	◯	◯	◯
Worsening pattern of existing headaches	◯	◯	◯
Abrupt or split second onset of severe headache	◯	◯	◯
Headache triggered by valsalva, cough or exertion	◯	◯	◯
Headaches developing during pregnancy	◯	◯	◯
Headaches first beginning after the age of 50 years	◯	◯	◯
Headaches associated with neurological signs (seizures, confusion, weakness papilledema)	◯	◯	◯
Child with headaches associated with systemic fever and neck stiffness	◯	◯	◯

ACORN
Australian Chiropractic Research Network

The management of headache disorders by Australian chiropractors

Chiropractic Headache Management

These questions are about your management of headaches.

* 24. How important are the following treatment outcomes to your management of patients with headache?

	Very unimportant,	Somewhat unimportant	Neutral	Somewhat important	Very important
Prevent headache episodes	○	○	○	○	○
Improve recovery from an episode of headaches	○	○	○	○	○
Pain relief during the headache episode	○	○	○	○	○
Improve headache-related coping skills	○	○	○	○	○
Improve overall health and well-being	○	○	○	○	○

ACORN
Australian Chiropractic Research Network

The management of headache disorders by Australian chiropractors

Chiropractic Headache Management

* 25. How frequently do you use the following treatment options in your management of patients with MIGRAINE?

	Never	Rarely	Often	Almost every migraine patient
Manual adjusting/ manipulation (including Diversified, Gonstead)	○	○	○	○
Non-thrust spinal mobilisations	○	○	○	○
Instrument adjusting	○	○	○	○
Drop piece, Thompson or similar	○	○	○	○
Massage, myofascial technique, stretching or trigger-points to neck/shoulder area	○	○	○	○
Electro-physical therapies (TENS, ultrasound etc)	○	○	○	○
Soft tissue or exercise therapy to temporo-mandibular region	○	○	○	○
Prescriptive exercises for the neck/shoulder region	○	○	○	○
Advice on stress management	○	○	○	○
Advice on diet or fitness	○	○	○	○
Advice on Headache triggers	○	○	○	○

Other therapies used for migraine (please specify):

ACORN
Australian Chiropractic Research Network

The management of headache disorders by Australian chiropractors

Chiropractic Headache Management

* 26. How frequently do you use the following treatment options in your management of patients with TENSION HEADACHE?

	Never	Rarely	Often	Almost every patient with tension headache
Manual adjusting/ manipulation (including Diversified, Gonstead)	○	○	○	○
Non-thrust spinal mobilisations	○	○	○	○
Instrument adjusting	○	○	○	○
Drop piece, Thompson or similar	○	○	○	○
Massage, myofascial technique, stretching or trigger-points to neck/shoulder area	○	○	○	○
Electro-physical therapies (TENS, ultrasound etc)	○	○	○	○
Soft tissue or exercise therapy to temporo-mandibular region	○	○	○	○
Prescriptive exercises for the neck/shoulder region	○	○	○	○
Headache triggers advice	○	○	○	○
Stress management advice	○	○	○	○
Diet or fitness advice	○	○	○	○

Other therapies used for tension headache (please specify):

ACORN
Australian Chiropractic Research Network

The management of headache disorders by Australian chiropractors

Chiropractic Headache Management

* 27. How frequently do you use the following treatment options in your management of patients with CERVICOGENIC HEADACHE?

	Never	Rarely	Often	Almost every patient with cervicogenic headache
Manual adjusting/ manipulation (including Diversified, Gonstead)	○	○	○	○
Non-thrust spinal mobilisations	○	○	○	○
Instrument adjusting	○	○	○	○
Drop piece, Thompson or similar	○	○	○	○
Massage, myofascial technique, stretching or trigger-points to neck/shoulder area.	○	○	○	○
Electro-physical therapies (TENS, ultrasound etc)	○	○	○	○
Soft tissue or exercise therapy to temporo-mandibular region	○	○	○	○
Prescriptive exercises for the neck/shoulder region	○	○	○	○
Stress management advice	○	○	○	○
Diet or fitness advice	○	○	○	○
Headache triggers advice	○	○	○	○

Other therapies used for cervicogenic headache (please specify):

ACORN
Australian Chiropractic Research Network

The management of headache disorders by Australian chiropractors

Chiropractic Headache Management

The following questions are about your headache treatment plan.

* 28. Indicate the average NUMBER of visits you provide during the initial period of care for a new patient presenting with a chief complaint of headache as listed below.

	Less than 5 treatments	5 to 10 treatments	More than 10 treatments
Migraine (less than 3 months duration)	○	○	○
Tension-type headache (less than 3 months duration)	○	○	○
Cervicogenic headache (less than 3 months duration)	○	○	○
Migraine (more than 3 months duration)	○	○	○
Tension headache (more than 3 months duration)	○	○	○
Cervicogenic headache (more than 3 months duration)	○	○	○

* 29. Indicate the average DURATION of the initial period of care for a new patient presenting with a chief complaint of headache as listed below.

	Less than 2 weeks	2-4 weeks	4-8 weeks	More than 8 weeks
Migraine, Tension-type headache or Cervicogenic headache (less than 3 months duration)	○	○	○	○
Migraine, Tension-type headache or Cervicogenic headache (more than 3 months duration)	○	○	○	○

* 30. Indicate the average FREQUENCY of visits for the initial period of care for a new patient presenting with a chief complaint of headache as listed below.

	Once per week	Twice per week	Three times per week	More than three times per week
Migraine, Tension-type headache or Cervicogenic headache (less than 3 months duration)	○	○	○	○
Migraine, Tension-type headache or Cervicogenic headache (more than 3 months duration)	○	○	○	○

ACORN
Australian Chiropractic Research Network

The management of headache disorders by Australian chiropractors

Chiropractic Headache Management

This final question is about your treatment results.

* 31. How effective do you perceive your chiropractic management to be for each of the following headache types?

	Never helps	Rarely helps	Sometimes helps	Often helps	Difficult to say
Migraine (less than 3 months duration)	○	○	○	○	○
Tension headache (less than 3 months duration)	○	○	○	○	○
Cervicogenic headache (less than 3 months duration)	○	○	○	○	○
Migraine (more than 3 months duration)	○	○	○	○	○
Tension headache (more than 3 months duration)	○	○	○	○	○
Cervicogenic headache (more than 3 months duration)	○	○	○	○	○
